# Characterization of Mce4A protein of *Mycobacterium tuberculosis*: role in invasion and survival

**DOI:** 10.1186/1471-2180-8-200

**Published:** 2008-11-19

**Authors:** Neeraj Kumar Saini, Monika Sharma, Amita Chandolia, Rashmi Pasricha, Vani Brahmachari, Mridula Bose

**Affiliations:** 1 Department of Microbiology, Vallabhbhai Patel Chest Institute, University of Delhi, Delhi, India; 2 Ambedkar Center for Biomedical Research, University of Delhi, Delhi, India

## Abstract

**Background:**

The *mce4 *operon is one of the four homologues of mammalian cell entry (*mce*) operons of *Mycobacterium tuberculosis*. The *mce4A *(Rv3499c) gene within this operon is homologous to *mce1A *(Rv0169), that has a role in host cell invasion by *M. tuberculosis*. Our earlier reports show that *mce4 *operon is expressed during the stationary phase of growth of the bacillus in culture and during the course of infection in mammalian hosts. *M. tuberculosis *carrying mutation in *mce4 *operon shows growth defect and reduced survival in infected mice. However, the intracellular localization of Mce4A protein and its direct role in cell entry or survival of the bacillus has not been demonstrated so far.

**Results:**

By transmission electron microscopy we have demonstrated that recombinant Mce4A protein facilitates the invasion of non-pathogenic strain of *E. coli *into non-phagocytic HeLa cells. We observe that *mce4A *gene has a role comparable to *mce1A *in the survival of recombinant *E. coli *in human macrophages. Using antibodies raised against Mce4A protein, we show that the protein is localized in the cell wall fraction of *M. tuberculosis *H37Rv stationary phase culture only.

**Conclusion:**

Mce4A protein is expressed during the stationary phase of broth culture and localizes in the cell wall fraction of *M. tuberculosis*. Mce4A protein expressed in non-pathogenic *E. coli *enables it to enter and survive within HeLa cells and the macrophages. As Mce4A protein is expressed during later phase of mycobacterial growth, our results raise the possibility of it playing a role in maintenance of persistent tubercular infection.

## Background

The World Health Organization has estimated that nearly one-third of the world's population is now latently infected with *Mycobacterium tuberculosis *and 8–10 million people develop active disease resulting in 2 million deaths each year [1,2]. The prevalence of this infection is largely due to the extended dormancy of *M. tuberculosis *in the host and its ability to cause disease even in the face of a highly orchestrated host immune response [3].

*M. tuberculosis *is believed to primarily invade and replicate within alveolar macrophages [4]. It has the ability to enter A549 cells of alveolar epithelial origin in culture [5] and also invade other non-phagocytic cells [6,7].

Role of Mce1A protein in the entry and survival of the pathogen is demonstrated using recombinant *E. coli *carrying cloned copy of the gene as well as latex microspheres coated with recombinant Mce1A protein, both of which were able to enter HeLa cells [8,9]. Additionally, it has been demonstrated that the recombinant *E. coli *expressing Mce1A can survive longer in these cells [8]. The continued analysis of the four *mce *operons of *M. tuberculosis *H37Rv genome has shown significant conservation of the protein sequences in the four operons [10]. The redundancy of these operons is explained by functional significance of the different *mce *operons of *M. tuberculosis *H37Rv by various studies including ours [11-15]. Earlier we reported the expression of *mce4 *and absence of *mce1 *transcripts in the specific tissues of both infected rabbits and guinea pigs during advanced disease conditions, which suggested that *mce4 *operon, may have a role in the long term survival of *M. tuberculosis *in host's tissue [11]. The survival mechanism of dormant tubercle bacilli is still under intense research [16-18]. It is suggested that mycobacterial persistence may rise as a response to environmental stress, such as reduced oxygen concentration within a host tissue [19]. It is demonstrated that deletion mutants of *mce3 *and *mce4 *operons of *M. tuberculosis *H37Rv are attenuated in mice [13]. By deleting eight genes and the first 250 bp of the ninth gene from *mce4 *operon it was shown that survival of *M. tuberculosis *was significantly reduced in mice [13]. However, direct involvement of individual proteins from *mce4 *operon in the survival or uptake of *M. tuberculosis *into the mammalian cells was not demonstrated.

In the present study we validate the role of Mce4A protein of *mce4 *operon not only in cell invasion but also in survival of the pathogen in human macrophages using recombinant *E. coli *expressing cloned *mce4A *gene. The cellular localization and *in vitro *expression of the Mce4A protein in *M. tuberculosis *was also analyzed.

## Methods

### Bacterial strains, cells and culture conditions

*M. tuberculosis *H37Rv was grown as shake-cultures in Middlebrook 7H9 broth (Difco) supplemented with OADC (oleic acid, albumin [bovine, fraction V], dextrose, catalase [Difco]) and 2% glycerol at 37°C.

*E. coli *DH5α and BL-21 (DE3) cells were grown in Luria-Bertani (LB) broth or on LB agar in presence of kanamycin (25 μg/ml) where ever appropriate. The human epithelial cell line HeLa cells were grown in complete Dulbecco's modified Eagle's medium (DMEM, Gibco) supplemented with 10 mM sodium pyruvate (Sigma), 10% FBS (fetal bovine serum, Gibco), HEPES (Sigma) and 10 μg of penicillin-streptomycin/ml (Sigma). THP-1 monocyte cell line was cultured in RPMI 1640 (Sigma) supplemented with 10% FCS (fetal calf serum, Gibco) and 2 mM L-glutamine (Sigma). Both the cell lines were grown at 37°C in presence of 5% CO_2_. PMA (phorbol myristate acetate, Sigma) was used for the adhesion of THP-1 cells.

### Cloning, expression and purification of Mce1A and Mce4A

Genomic DNA from *M. tuberculosis *H37Rv was extracted according to the CTAB (cetyltrimethylammonium bromide) method [20]. Full length *mce1A *(Rv0169, 1365 bp) and *mce4A *(Rv3499c, 1203 bp) genes were amplified from *M. tuberculosis *genomic DNA [21] by polymerase chain reaction (PCR) using the primers mce1F and mce1R for *mce1A *gene, mce4F and mce4R for *mce4A *gene as listed in Table 1. Each forward primer contained *Sac*I restriction enzyme site whereas the reverse primers contained a *Hind*III restriction site.

**Table 1:** Primers used for PCR amplification of *mce1A *and *mce4A *gene.

Primer*	Sequence (5' to 3')	Gene
mce1F	ATCTCACGGTGT**GAGCTC**ATGACGACGCCGG	*mce1A*
mce1R	CGACGG TTCCAG**AAGCTT**TCATGGGTTGAT	
mce4F	CTTCAGAAA**GAGCTC**ATGTCCGGCGGCGGA	*mce4A*
mce4R	CGCCCGAGC**AAGCTT**TCAGAAGTCGTCCCG	

* Restriction sites in primers are indicated in bold letters.

The amplicons were cloned in pET28a (Novagen) to generate pET28a/*mce1A *and pET28a/*mce4A *with hexa histidine tags. Both DH5α and BL-21 (DE3) strains of *E. coli *were transformed with the recombinant plasmids for expression studies. The presence of the inserts was confirmed by restriction enzyme digestion and sequencing.

Overnight grown culture of *E. coli *BL-21 (DE3) containing the recombinant expression plasmids was diluted 1:10 in fresh LB broth containing kanamycin and grown with vigorous shaking at 37°C to an optical density (OD) of 0.6 at 600 nm. For transcription induction, IPTG (isopropyl thio-b-D-galactoside, Sigma) to a final concentration of 0.5 mM was added to the culture and incubation was continued for 2 h. The N-terminal His-tagged recombinant proteins were purified under denaturing condition on nitrilotriacetic acid (Ni-NTA) agarose columns (Qiagen) according to the manufacturer's instructions.

### Production of polyclonal antibodies against Mce4A protein

The antibodies against the Mce4A protein were raised in 5-month-old female NZW rabbit. For each immunization, 300 μg of protein in 0.5 ml PBS was mixed with 0.6 ml of incomplete Freund's adjuvant (Sigma) and administered subcutaneously at five sites in rabbit, followed by four booster injections of 300 μg each 2, 3, 4 and 6 weeks after the first injection. Serum was prepared from blood collected 2 weeks after the last booster dose. The animal handling was according to the guidelines of the Institutional Ethical Committee and Indian Council of Medical Research, India.

### Preparation of subcellular fractions

*M. tuberculosis *H37Rv was grown in one liter of 7H9 Middlebrook medium to mid-log phase (day 6) at 37°C on shaking (200 rpm) up to an OD of 0.6 at 600 nm. Cells were harvested by centrifugation at 10,000 g for 30 min and the culture supernatant was concentrated to obtain the culture filtrate protein (CFP). Pelleted cells were washed, resuspended in PBS and subcellular fractions were prepared as previously described [22,23]. Similarly, cellular fractions were also prepared from the stationary phase culture (day 10, OD of 1.0 at 600 nm) of *M. tuberculosis *H37Rv.

### Detection of protein expression

The protocol followed for Western blotting of purified Mce1A and Mce4A proteins was as described previously [24]. Anti-His monoclonal antibody (1:2000 dilution) and peroxidase-conjugated goat anti-mouse IgG (1:2000) were used as primary and secondary antibody respectively. Western blot was carried out to detect purified Mce4A protein and also the expression of Mce4A protein in the whole cell lysate of cells harvested on day 2 (OD-0.2 at 600 nm), day 4 (OD-0.4), day 7 (OD-0.7), and day 10 (stationary phase, OD-1.0) post inoculation. Polyclonal anti-Mce4A antibody (1:2000) raised in rabbits, was used as the primary antibody. For the intracellular localization of Mce4A protein in *M. tuberculosis*, Western blotting was performed with cytoplasmic (C), cell membrane (CM), cell wall (CW) fractions, culture filtrate protein (CFP) and whole cell lysate (CL) prepared from both log and stationary phase culture as described above.

### Invasion assay and Electron microscopy

Invasion of HeLa cells seeded at 2 × 10^6 ^cells per well into 6-well plates by BL-21 cells transformed with pET28a/*mce1A*, pET28a/*mce4A *and empty vector pET28a was assayed. Bacterial cultures grown overnight were diluted (1:10) in fresh medium containing kanamycin and incubated further with vigorous shaking to reach an OD of 0.6 at 600 nm and induced with 0.5 mM IPTG for 2 h at 37°C. Following the induction, the cells were washed once with PBS and resuspended in 5 ml of PBS. Prior to the infection, monolayer of HeLa cells were charged with fresh medium (DMEM, supplemented with 10% FCS) and incubated at 37°C for 30 min. Recombinant *E. coli *cells were added to the monolayer at a multiplicity of infection (MOI) of 10:1 and incubated at 37°C for 4 h, washed three times with PBS and processed for electron microscopy as described previously [9] and examined under CM-10 electron microscope. Since the *E. coli *strain used is kanamycin resistant, the cells were not treated with kanamycin or gentamycin but were extensively washed post-infection to remove the extra-cellular bacteria that failed to enter the mammalian cells.

### Survival assay

THP-1 cells were seeded at 2 × 10^6 ^cells per well into 12-well plates. PMA (20 ng/ml) was added for the cell adhesion. Recombinant bacterial cultures induced with IPTG were incubated with THP-1 cells at a MOI of 10:1. Prior to the infection, THP-1 cells were charged with fresh medium (RPMI supplemented with 10% FCS and 2 mM L-glutamine) and incubated at 37°C for 30 min. Infection was continued for 1 h at 37°C.

Following 1 h incubation, the cells in all the wells were washed three times with FCS free RPMI. One set of cells were lysed immediately after 1 h of incubation. The CFU at this time point was taken as t_0 _(CFU at time 0). The other sets were lysed after 3 h (t_3_) and 24 h (t_24_) of incubation. Briefly, the cells were incubated for 10 min in 500 μl of lysis buffer (0.1% Triton X-100 in PBS, pH 7.4). The lysate was plated on LB agar plates and incubated at 37°C overnight and recombinant *E. coli *colonies were counted. The numbers of *E. coli *able to survive at 3 h and 24 h post infection were normalized against CFU at t_0 _and expressed as percentage survival. Experiment was done in triplicate.

### Statistical analysis

The CFU of recombinant *E. coli *and the percentage survival of recombinant *E. coli *cells are represented as mean ± standard deviation. The difference in percentage survival of recombinant *E. coli *cells (pET28a/*mce1A *and pET28a/*mce4A*) as compared to *E. coli *(pET28a) was calculated using student's t test using graph pad prism^Tm ^software.

## Results

### Cloning, expression and purification of Mce1A and Mce4A proteins

**Figure 1:**
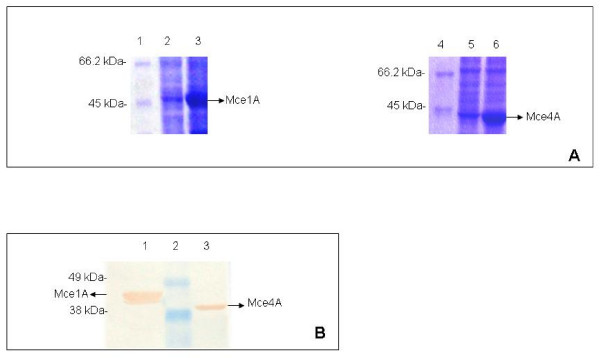
**SDS-polyacrylamide gel electrophoresis and Immunoblot analysis**. (A) SDS-polyacrylamide gel electrophoresis of whole-cell lysates of uninduced and induced recombinant *E. coli *cells. Lane 1, 4- Molecular mass marker (116 kDa, 66.2, 45, 35, 25, 18.4, 14.4, Fermentas), the molecular mass standards are indicated in kDa on left side. Lane 2, 3- Lysate of uninduced and induced *E. coli *(pET28a/*mce1A*) cells respectively. Lane 5, 6- Lysate of uninduced and induced *E. coli *(pET28a/*mce4A*) cells respectively. Mce1A and Mce4A are indicated by arrows. (B) Immunoblot of purified Mce1A and Mce4A proteins. Anti-His-tag antibody was used to demonstrate the fusion proteins. Lane1-Purified Mce1A protein (2 μg). Lane 2- Pre-stained molecular mass marker (188 kDa, 62, 49, 38, 28, 18, 14, 6, 3, Invitrogen), the molecular mass standards in kDa are indicated on left side. Lane 3-Purified Mce4A protein (2 μg). Mce1A and Mce4A are indicated by arrows.

**Figure 2:**
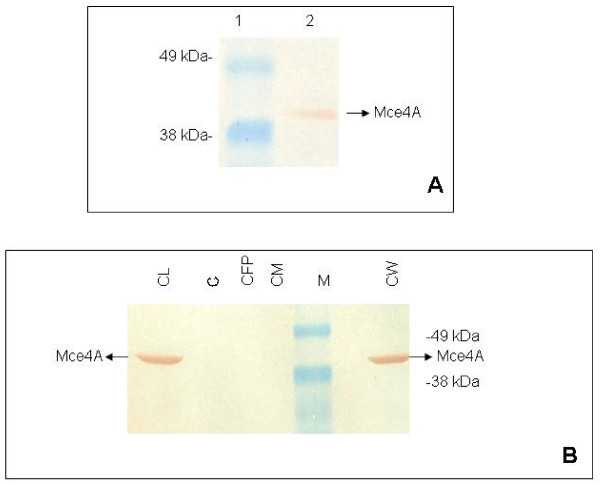
**Immunoblot analysis of Mce4A protein**. (A) Purified Mce4A protein separated by SDS-PAGE and electrotransferred to nitrocellulose membrane. Rabbit polyclonal antibody to Mce4A protein used at a dilution of 1:2000, detected signal of purified Mce4A protein. Lane 1-Pre-stained molecular mass marker (188 kDa, 62, 49, 38, 28, 18, 14, 6, 3, Invitrogen), the molecular mass standards are indicated in kDa on left side. Lane 2: Purified Mce4A protein (1 μg). Mce4A protein is indicated by arrow. (B) Subcellular fractions of *M. tuberculosis *H37Rv, stationary phase culture were fractionated on SDS-PAGE and Western blotting was performed using polyclonal anti-Mce4A antibodies for localization of Mce4A in various fractions. Concentration of protein loaded per well is indicated in parenthesis. Lane CL-Whole cell lysate (50 μg), Lane C-Cytoplasm (40 μg), Lane CFP-Culture filtrate proteins (30 μg), Lane CM-Cytoplasmic membrane (30 μg), Lane M-Pre-stained molecular mass marker (188 kDa, 62, 49, 38, 28, 18, 14, 6, 3, Invitrogen), the molecular mass standards are indicated in kDa on right side, Lane CW-Cell wall fraction (30 μg). Mce4A protein is detected only in the whole cell lysate and cell wall fraction from stationary phase culture.

The expression of the fusion proteins was studied in *E. coli *BL-21 (DE3) strain. The His-tagged Mce1A and Mce4A were expressed at high level on induction with IPTG and the purified proteins were detected using anti-His-tag monoclonal antibody (Fig. 1B). The fusion proteins were of the expected size of 45 kDa and 43 kDa respectively (Fig. 1A). Further we confirmed the identity of purified Mce4A protein with rabbit anti-Mce4A polyclonal antibodies raised in our laboratory (Fig. 2A). Mce4A was purified as a single band, while Mce1A was detected as a doublet. Since anti-His-tag antibodies detected the lower molecular weight band, it is probably due to partial degradation. Similar profile for Mce1A protein was reported earlier also [25].

### Growth-phase dependent expression and subcellular localization of Mce4A protein of *M. tuberculosis*

Immunochemical detection of Mce4A protein in whole cell lysate from different phase of *M. tuberculosis *cultures shows that it is expressed only in stationary phase culture (data not shown) and the subcellular fractions from stationary phase culture show that Mce4A is localized in cell wall fraction only (Fig. 2B). In a similar experiment subcellular fraction from mid-log phase culture of *M. tuberculosis *H37Rv, antibodies against Mce4A did not detect any protein either in whole cell lysate or any subcellular fraction (data not shown), which indicates absence of expression of Mce4A during early phase of growth.

### Invasion of HeLa cells by recombinant *E. coli *expressing Mce4A protein

**Figure 3:**
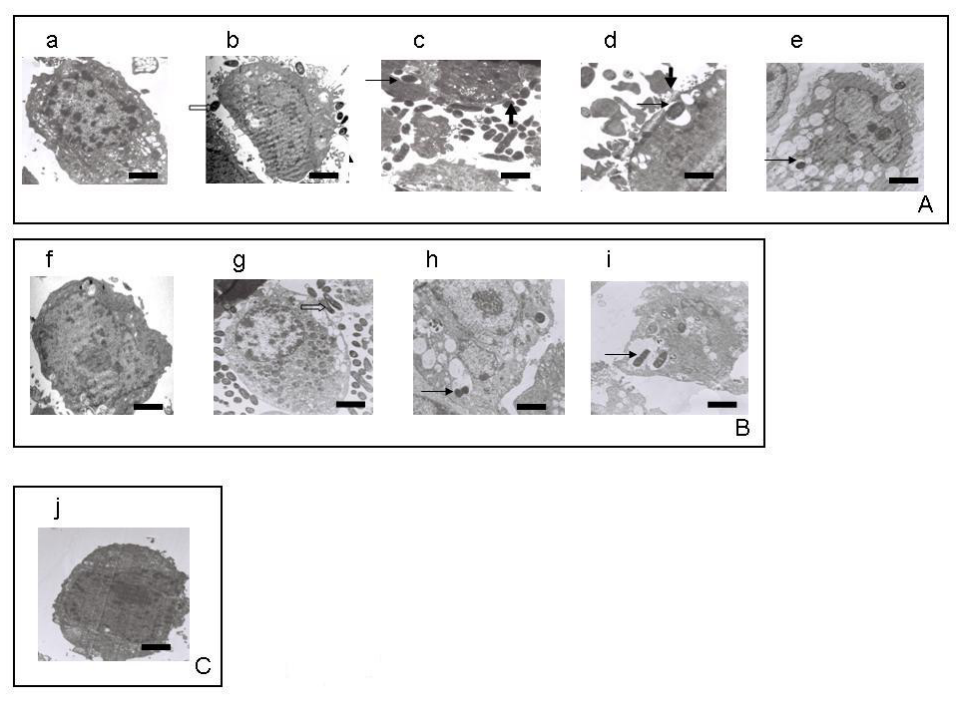
**Invasion of HeLa cells by recombinant *E. coli***. The invasion of HeLa cells by *E. coli *expressing Mce4A and Mce1A proteins was monitored by transmission electron microscopy. The stages of invasion by recombinant *E. coli *(pET28a/*mce4A*) (fig. 3A), *E. coli *(pET28a/*mce1A*) (fig. 3B) and *E. coli *(pET28a) (fig. 3C: j) are shown. A: a and B: f infection with uninduced recombinant *E. coli*. A: b, c, d, e and B: g, h, i infection with induced recombinant *E. coli *expressing Mce4A and Mce1A respectively. Unfilled arrow (bacteria attached to cell membrane), thick arrow (membrane ruffling), thin arrow (internalized bacteria). Bars = 1 μm in all the cases, except in A: c, d and B: g, h, where it is 0.1 μm.

The invasion of HeLa cells by recombinant *E. coli *was monitored by transmission electron microscopy. The results show that uninduced *E. coli *(pET28a/*mce4A *and pET28a/*mce1A*) (Fig. 3A: a and 3B: f respectively) were not able to enter the HeLa cells while IPTG induced recombinant *E. coli *expressing Mce4A protein (Fig. 3A: c–e) and Mce1A protein (Fig. 3B: h–i) were able to invade the HeLa cells. The invaded HeLa cells showed membrane-ruffling, presence of multiple invaginations at the cell membrane and multiple vacuolations in the cells (Fig. 3A: b–e and 3B: g, i). Internalized recombinant *E. coli *were observed within the cytoplasmic vacuoles (Fig. 3A: c–e and 3B: h–i). The *E. coli *(pET28a) used as a negative control failed to enter the HeLa cells (Fig. 3C: j).

### Role of Mce4A protein in survival of *E. coli *in the THP-1 macrophages

To determine the role of Mce4A protein in survival of *M. tuberculosis *within macrophages, the THP-1 cells were infected with recombinant *E. coli *expressing Mce4A or Mce1A at MOI of 10:1 and percentage survival was determined at 3 h and 24 h post-infection. The percentage survival at 3 h post-infection of *E. coli *(pET28a/*mce1A*) and *E. coli *(pET28a/*mce4A*) was significantly higher than that of *E. coli *(pET28a) at the same time points post-infection (p < 0.005) (Table 2). At 24 h post-infection, negligible number of colonies of *E. coli *(pET28a) was obtained from lysed cells as compared to that from *E. coli *(pET28a/*mce1A*, p < 0.0005) and *E. coli *(pET28a/*mce4A *p < 0.005) (Table 2). At 3 h and 24 h percentage survival of *E. coli *(pET28a/*mce4A*) was comparable to that of *E. coli *(pET28a/*mce1A*) (p > 0.05 at 3 h and 24 h).

**Table 2:** Survival of recombinant *E. coli *within THP-1 cells

Recombinant *E. coli *used to infect THP-1 cells	Colony forming units at different time points (% survival)		
	t_0_	t_3_	t_24_
	
Vector only	1426 ± 112	376 ± 37.74 (26.3 ± 1.8)	6.6 ± 1.45 (0.46 ± 0.12)
pET28a/*mce4A*	3157 ± 37.17	1493 ± 90.07 (47.2 ± 2.7^φφ^)	556 ± 60.80 (18.3 ± 2.17^θθ^)
pET28a/*mce1A*	3596 ± 106.4	2209 ± 146.3 (61.5 ± 4.59**)	770.7 ± 59.05 (21.33 ± 1.09***)

Survival was assayed as colony forming units per milliliter of lysate following lysis of the infected cells. Time points considered were as follows: t_0 _- 1 hour, t_3 _- 3 hours and t_24 _- 24 hours, post-infection. All values were normalised to t_0 _value taken as 100%. The significance was calculated using t test. Recombinant *E. coli *expressing Mce4A or Mce1A survived inside THP-1 cells at a significantly high CFU as compared to recombinant *E. coli *with vector alone.

^φφ^, p < 0.005; **, p < 0.005; when t test applied to compare percentage survival of *E. coli *(pET28a) with percentage survival of *E. coli *(pET28a/*mce4A*) and *E. coli *(pET28a/*mce1A*) respectively at 3 h of incubation.

^θθ^, p < 0.005; ***, p < 0.0005; t test applied to compare percentage survival of *E. coli *(pET28a) with percentage survival of *E. coli *(pET28a/*mce4A*) and *E. coli *(pET28a/*mce1A*) respectively at 24 h of incubation.

## Discussion

The initial step in the pathogenesis by intracellular pathogens is the invasion of host cells. In case of *M. tuberculosis*, Mce1A protein of *mce1 *operon was the first protein of the pathogen to be implicated in invasion and survival inside macrophages [8]. Although Mce2A protein of *mce2 *operon exhibits 67% amino acid identity with Mce1A, latex beads coated with Mce2A are not internalized by HeLa cells [9]. In the present study we have demonstrated that recombinant *E. coli *expressing Mce4A protein can enter non-phagocytic HeLa cells. Further, Mce4A protein also enables the recombinant *E. coli *to survive inside THP-1 macrophages. The estimation of CFU demonstrates that the rate of intracellular survival of recombinant *E. coli *expressing *mce4A *was comparable to that of *mce1A *indicating thereby that *mce4A *may have a role similar to *mce1A *in invasion and survival of *M. tuberculosis *inside the host cells.

Molecular modeling studies using bioinformatics tools have shown that cell entry epitope is exposed on the surface of Mce1A, Mce3A and Mce4A proteins but not on the Mce2A protein [26,27]. This suggests that the cell entry epitope of Mce1A, Mce3A and Mce4A, but not of Mce2A, may be available for interaction with mammalian cells. Subsequently a report appeared confirming the role of Mce3A and Mce3E in mammalian cell entry [12].

The recently emerging reports on the role of *mce4 *operon in survival of *M. tuberculosis *in host tissue indicates that Mce4 proteins may be involved in maintaining the *M. tuberculosis *in a nutrient deficient environment for long term survival [14]. It has been suggested that the proteins of the *mce4 *operon may operate as a major cholesterol import system of *M. tuberculosis *because strains lacking *mce4 *operon exhibit drastically reduced ability to take up and metabolize cholesterol *in vitro *and hence grow poorly when cholesterol is the primary source of carbon [14]. It was reported earlier that mutation in *mce1 *operon produces growth defect in early phase of infection in mice and mutation in *mce4 *operon produces growth defect after 3–4 weeks post infection, indicating thereby that *mce4 *operon is required in later phase of infection [28].

The localization study showed that the Mce4A protein was present in the cell wall fraction of the stationary phase culture of *M. tuberculosis *H37Rv further strengthening our results of transmission electron microscopy and cell invasion studies.

We conclude that the results of the present study strongly support the role of Mce4A protein in invasion and the intracellular survival of *M. tuberculosis *and suggest that it may have a role in persistence of mycobacterial infection in the host tissue.

## Conclusion

There are many studies reported in the literature to elucidate dormancy in *M. tuberculosis *and the survival mechanism of dormant tubercle bacilli is not fully understood. It is suggested that mycobacterial persistence might arise as a response to environmental stress, such as reduced oxygen concentration within host tissue. Our earlier observation of preferential expression of *mce4 *operon in the internal organs of rabbit and guinea pig and also during the stationary phase in culture in conjunction with the results presented here strongly support a role for Mce4A protein in the long term survival of *M. tuberculosis *inside its host.

## Authors' contributions

MB conceived the study. MB, VB and NKS designed the experiments, interpreted the results and worked on the manuscript. NKS carried out major experimental work. MS was associated with tissues culture studies. AC and RP participated in the designing of the experiments and the interpretation of the results. All authors read and approved the final manuscript.
